# The Biochemical and Functional Characterization of M28 Aminopeptidase Protein Secreted by *Acanthamoeba* spp. on Host Cell Interaction

**DOI:** 10.3390/molecules24244573

**Published:** 2019-12-13

**Authors:** Jian-Ming Huang, Yao-Tsung Chang, Wei-Chen Lin

**Affiliations:** 1 Institute of Basic Medical Sciences, College of Medicine, National Cheng Kung University, Tainan 701, Taiwan; et10005@hotmail.com; 2 Department of Biochemistry and Molecular Biology, College of Medicine, National Cheng Kung University, Tainan 701, Taiwan; ytchang.ncku@gmail.com; 3 Department of Parasitology, College of Medicine, National Cheng Kung University, Tainan 701, Taiwan; 4 Department of Microbiology and Immunology, College of Medicine, National Cheng Kung University, Tainan 701, Taiwan

**Keywords:** *Acanthamoeba* spp., secreted protein, M28AP, apoptosis

## Abstract

*Acanthamoeba* are a free-living protozoan whose pathogenic strain can cause severe human diseases, such as granulomatous encephalitis and keratitis. As such, the pathogenic mechanism between humans and *Acanthamoeba* is still unknown. In our previous study, we identified the secreted *Acanthamoeba* M28 aminopeptidase (M28AP) and then suggested that M28AP can degrade human C3b and iC3b for inhibiting the destruction of *Acanthamoeba* spp. with the human immune response. We constructed the produced the recombinant M28AP from a CHO cell, which is a mammalian expression system, to characterize the biochemical properties of *Acanthamoeba* M28AP. The recombinant M28AP more rapidly hydrolyzed Leu-AMC than Arg-AMC and could be inhibited by EDTA treatment. We show that recombinant M28AP can be delivered into the individual cell line and cause cell line apoptosis in a co-culture model. In conclusion, we successfully investigated the potential molecular characteristics of M28AP.

## 1. Introduction

*Acanthamoeba* spp. is a free-living pathogenic protozoan that causes severe sight-threatening infection, such as granulomatous amoebic encephalitis (GAE) and Acanthamoeba keratitis [1,2]. *Acanthamoeba* spp. is widely distributed in the environment, including in lakes, pools, soil, and dust [3]. The *Acanthamoeba* spp. life cycle includes both motile trophozoites and dormant cysts. The trophozoite feeds on a variety of organic materials, such as bacteria via phagocytosis. The dormant cyst stage has double walls to resist and survive in harsh environmental conditions [4]. Free-living amoebae, such as *Naegleria* spp., *Sappinia* spp., *Acanthamoeba* spp., and *Balamuthia* spp., are known to cause fatal human central nervous system (CNS) infections. Headache, fever, nausea, vomiting, stiff neck, photophobia, confusion, and death are included as symptoms [5]. *Acanthamoeba* spp. infecting the human CNS may result in GAE through the blood–brain barrier [6]. *Acanthamoeba* spp. causes the amoebae invasion of the alveolar blood vessels through the respiratory tract. Patients undergoing immunosuppressive therapy or excessively use steroids are at risk of GAE. A previous a case report showed that GAE was caused by *Acanthamoeba* spp. in a patient with kidney transplant [7].

Acanthamoeba keratitis (AK) is a rare and severe parasitic infection ocular infection that results from free-living pathogenic *Acanthamoeba* spp. [1,2]. However, AK has increased with the widespread use of contact lenses over the past two decades [3,8,9,10]. *Acanthamoeba* spp. infects patients wearing contact lenses over a long period of time through corneal injury [11]. Patients suffering from AK may experience lid edema, photophobia, epithelial defect, and ring-like stromal infiltrate [12]. AK has serious consequences, such as loss of vision if not treated adequately and immediately, due to difficulty in early diagnosis and poor response to most antibiotics [5,13,14,15,16]. Therefore, the mechanism through which the virulence factors of *Acanthamoeba* spp. cause tissue invasion or infection in these patients must be elucidated.

Previous studies reported that aminopeptidase could play important roles in infections. Secreted matrix metalloproteases are synthesized as proenzymes into the extracellular space and they can be activated by many mechanisms, including normal physiology and pathology during development, wound healing, and cancer cell metastasis [17,18]. In parasites, an aspartyl aminopeptidase-like gene has been identified in the *Toxoplasma gondii* genome that affects the growth of *T. gondii* via parasite replication and growth [19]. GP63, which is a parasite metalloprotease of *Leishmania* species, is known in the cleavage and degradation of the activity of the complement system to avoid the human immune system [20]. An M17 leucine aminopeptidase of *Acanthamoeba castellanii* plays an important role during the encystation of *Acanthamoeba* spp. [21]. *Acanthamoeba* spp. produces cysteine, serine, and metalloproteases, which are increased in pathogenic *Acanthamoeba* strains [22,23].

We previously showed that the M20/M25/M40 superfamily aminopeptidase protein, which is an aminopeptidase found in *Acanthamoeba*-secreted proteins (Asp), causes cell damage and disruption [24]. We also identified a novel protein that was secreted by *Acanthamoeba* spp., an M28 aminopeptidase (M28AP), as a target of the human innate immune defense [25,26]. However, little is known regarding the biochemical and functional characterization of M28AP. Aminopeptidases, which are also called metalloproteases, are a highly diverse set of proteolytic enzymes that catalyze the cleavage of amino acids. Aminopeptidases distribute across homologous protease families M1 through M91 [27]. Metalloprotease has a conserved HEXXH active site motif that is mediated by one or two divalent ions, often zinc. The conserved HEXXH active site motif activates the nucleophilic attack of water molecules on substrate peptide bonds [28]. A previous study showed that families of metalloproteases could be divided into five groups. The first set has a glutamic acid residue at the metal-binding site (HEXXH+E), such as M1, M2, M4, M5, and M13. The second set has a third histidine residue at the metal-binding site (HEXXH+H), such as M7, M10, M11, and M12. The third set has an additional metal-binding site (HEXXH) that is yet unidentified, such as M3, M6, M8, M9, M26, M27, M30, M31, and M12. The fourth group is bound at motifs other than HEXXH, for example, M14, M15, M16, M17, and M24. The final group of families is until unknown in the metal ligands, which includes M18, M19, M20, M22, M23, M25, M28, M29, and M33 [29]. In the present study, we found that recombinant M28AP that was purified from CHO cells had aminopeptidase activity to hydrolyze a highly sensitive substrate. As metalloprotease, the recombinant M28AP was also inhibited by EDTA treatment. In a co-culture model, the recombinant M28AP can be delivered into individual cell line and cause cell line apoptosis in vitro.

## 2. Results

### 2.1. The Virulence of Asp Induces Cell Damage in Human Primary Corneal Epithelial Cells

**Figure 1:**
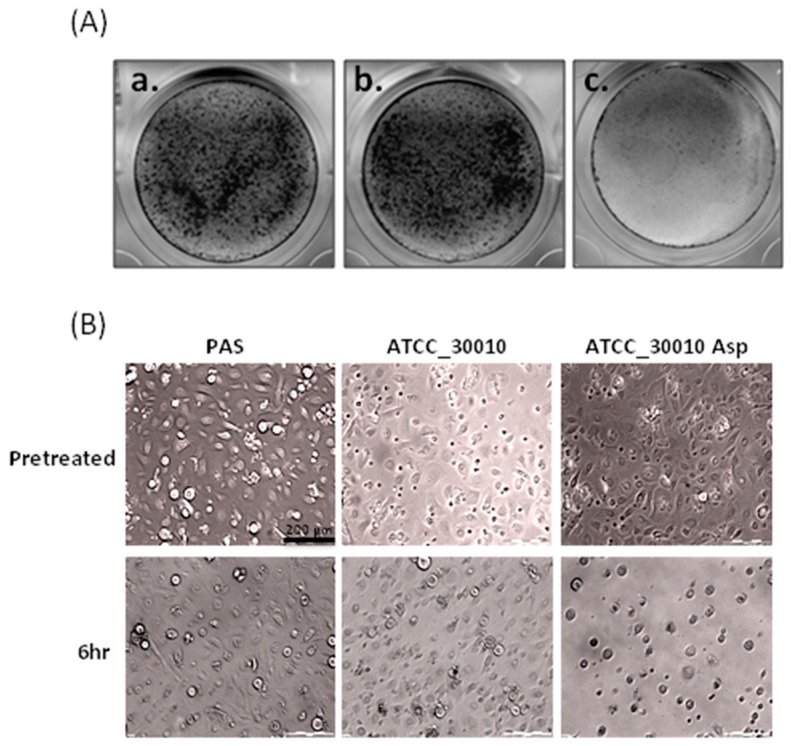
Cytopathic Effect Assay (CPE) functional assays of *Acanthamoeba* spp. and the Asp of *Acanthamoeba* spp. in human primary corneal epithelial cells. (**A**) The effect of *Acanthamoeba* spp. and the Asp of *Acanthamoeba* spp. in the cell line used for the Giemsa stain. The observation of the cell co-incubated with *Acanthamoeba* spp. for six hours. The cells were treated with (**a**) Page’s modified Neff’s amoeba saline (PAS), (**b**) *Acanthamoeba* spp., and (**c**) the Asp of *Acanthamoeba* spp. (**B**) The human primary corneal epithelial cells co-incubated with *Acanthamoeba* spp. and the Asp of *Acanthamoeba* spp. examined while using microscopy after six hours.

We chose a suitable strain and corrected the damage-induced duration for observation while using CPE assays to clarify the pathogenesis mechanisms of *Acanthamoeba* spp. The results show that no significant change occurred in the cell coverage area between the corneal epithelial cells that were co-incubated six hours with the *Acanthamoeba* spp. and control (mock) (Figure 1A_a,b). We isolated the Asp of *Acanthamoeba* spp. to treat the corneal epithelial cells while using a CPE assay to investigative whether Asp are involved the cell disruption process of *Acanthamoeba* spp. The results of the CPE assay showed that Asp of *Acanthamoeba* spp. could disrupt the co-incubated corneal epithelial cells (Figure 1A_c). We observed that Asp of *Acanthamoeba* spp. induced the loss of cell adhesion ability in a time-dependent manner via microscopy (Figure 1B). We suggested that Asp of *Acanthamoeba* spp. might have the important virulence factors to affect the cell line.

### 2.2. Biochemical Characterization of M28AP

**Figure 2:**
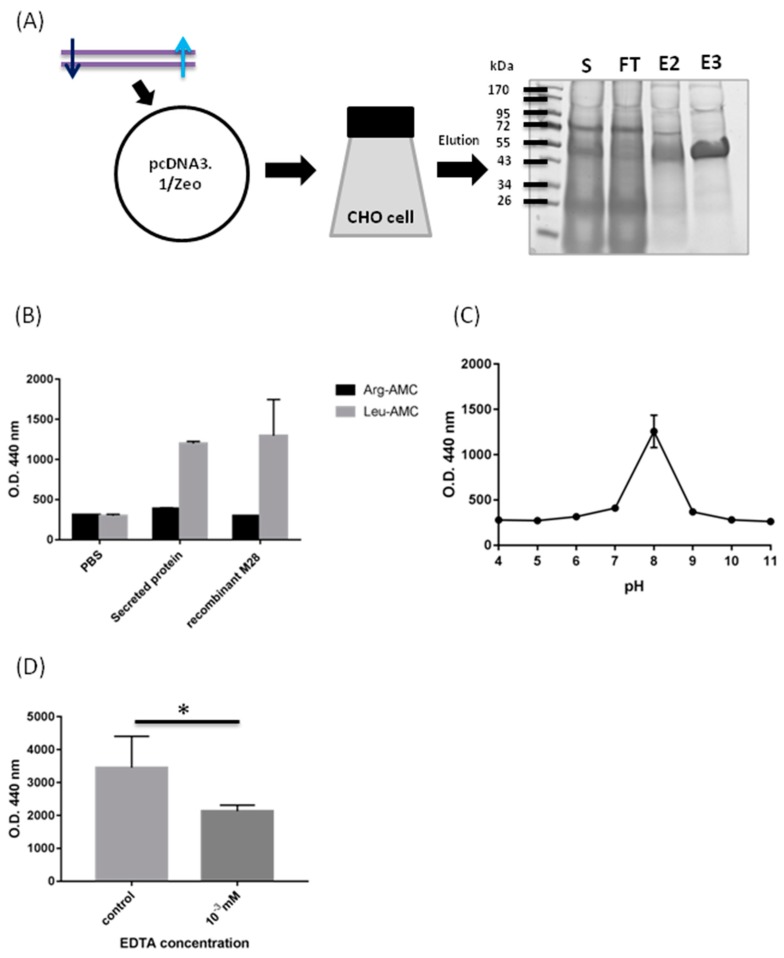
Characterization of the secreted *Acanthamoeba* M28 aminopeptidase (M28AP). (**A**) Recombinant M28AP protein expressed by pcDNA3.1/Zeo vector transformed Chinese hamster ovary (CHO) cell. The recombinant M28AP (arrows) was purified from CHO cells and stained with Coomassie Blue. (S: supernatant, FT: flow-through, E2-3: Eluted fractions of the recombinant M28AP protein through column) (**B**) Aminopeptidase activity of the Asp of *Acanthamoeba* spp. and the recombinant M28AP protein. (PBS was the negative control.) (**C**) Optimum pH of M28AP activity assayed at 37 °C for 30 min with Leu-AMC substrate. (**D**) Effects of metal chelators and EDTA on the activity of M28AP with Leu-AMC substrate. (* *p* ≤ 0.05).

In a previous study, we established the extracellular secreted proteomic database of *Acanthamoeba* spp. [25]. We found a novel protein secreted by *Acanthamoeba* spp., M28AP, from the extracellular secreted proteomic database as a target of the human innate immune defense system [26]. We purified the recombinant M28AP from CHO cells to characterize the biochemical properties of M28AP (Figure 2A). We used the recombinant M28AP and collected Asp of *Acanthamoeba* spp. that contained extracellular vesicles and soluble proteins to treat Leu-AMC and Arg-AMC to detect whether the recombinant M28AP from CHO cells is active. The results showed that Asp and the recombinant M28AP more rapidly hydrolyzed Leu-AMC than Arg-AMC, which a highly sensitive substrate for aminopeptidase (Figure 2B). The recombinant M28AP showed aminopeptidase activity in the approximately optimal pH range (pH 7.0–9.0), with a maximum at pH 8.0 (Figure 2C). Metal chelators and EDTA were used for the inhibition of M28AP enzymatic activity and stability, since M28AP is a metalloenzyme. The result showed that recombinant M28AP treated with EDTA at concentrations of 10^−3^ mM inhibited approximately 40% of the enzymatic activity when compared with the control (Figure 2D).

### 2.3. The Role of M28Ap as Virulence Factor and Host Cell Damage

**Figure 3:**
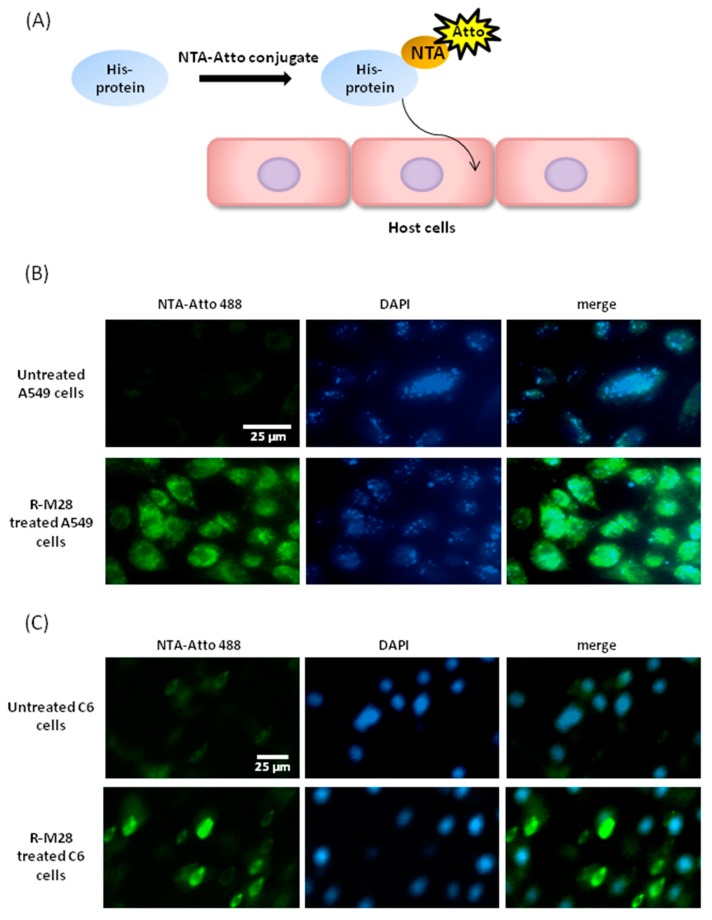
The interaction of M28AP with cell line by NTA-Atto conjugate. (**A**) A schematic model of NTA-Atto conjugate assay. (**B**,**C**) The interaction of recombinant M28AP with A549 and C6 cells with NTA-Atto conjugate for four hours. (Green: NTA-Atto conjugated M28AP; blue: DAPI).

We used NTA-Atto conjugated with his-tag protein, the recombinant M28AP, to observe M28AP host–cell interaction to determine whether the secreted protein, M28AP, was delivered into individual cell line (Figure 3A). In this study, we substituted the C6 and A549 cell lines for human primary corneal epithelial cells, because primary cells have a limited lifespan and are harder to culture than these cell lines. The results showed that the recombinant M28AP was delivered into the individual cell line and not only the C6 and A549 cells after four hours of incubation (Figure 3B,C). We suggest that the M28AP of *Acanthamoeba* spp. is secreted and delivered into cell line to cause cell damage.

**Figure 4:**
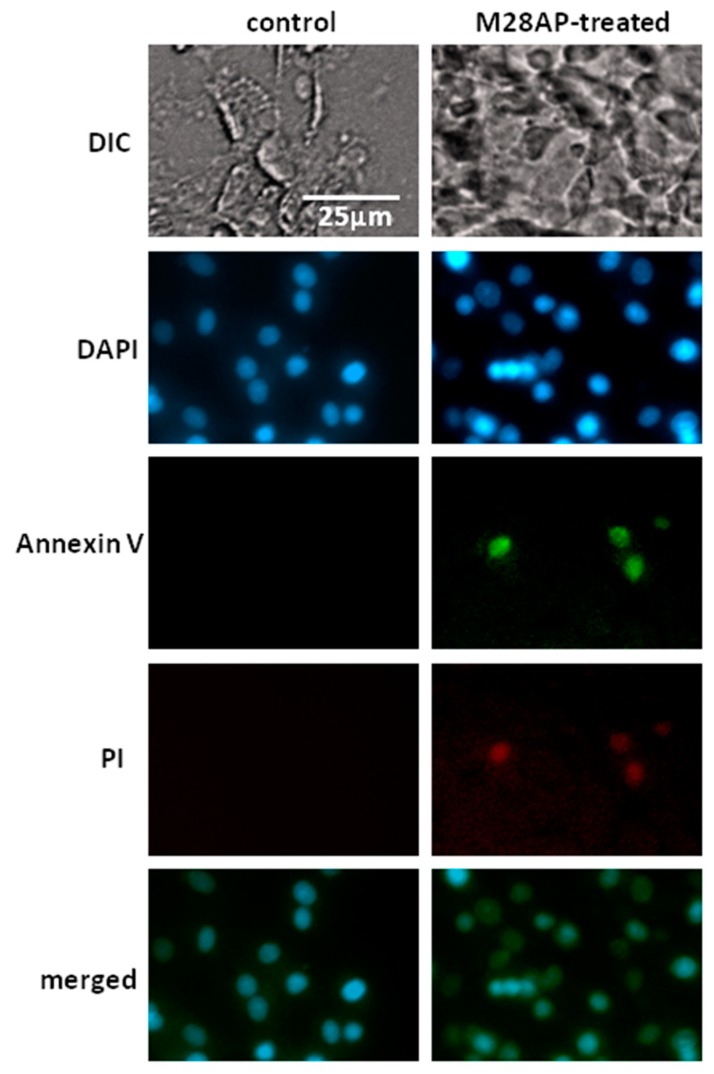
Annexin V apoptosis assay of cell line treated with recombinant M28AP. The Annexin V apoptosis assay of the C6 cells treated with the recombinant M28AP for 24 h were used for light and fluorescence microscopy. (Green, Annexin V; red, PI; blue, DAPI).

An Annexin V assay kit was used to detect apoptotic cells to investigative whether the M28AP of *Acanthamoeba* spp. causes cell line damage. The result showed that C6 cells that were treated with recombinant M28AP were both Annexin V and PI positive (Figure 4). Hence, we suggest that M28AP of *Acanthamoeba* spp. may act as a virulence factor to cause late cell apoptosis and cell death. However, from the results, we observed that M28AP caused minor apoptosis of C6 cells, although the apoptotic cells were detected while using the Annexin V assay. Therefore, we suggest that Asp contain many different proteases and M28AP might be a minor virulence factor that causes cell line damage via apoptotic pathways.

## 3. Discussion

### 3.1. Biochemical Characterization of M28AP

Aminopeptidases, which are also called metalloproteases, are a highly diverse set of proteolytic enzymes that catalyze the cleavage of amino acids and are distributed across homologous protease families M1 through M91 [27]. Metalloproteases cleave a peptide bond with a hydrophobic side chain, such as Leu, Ile, Met, Phe, or Tyr. These hydrophobic residues fit into the specificity pocket of the different aminopeptidase [30]. The matrix metalloproteases 2 and 7 of *Bacillus cereus* were cleaved with high efficiency at the Leu–Gly or Leu–Ala bond by the residue in the P1′ position [31]. A previous study showed that the M20/M25/M40 superfamily aminopeptidase protein, an aminopeptidase found in Asp can be hydrolyzed Leu-AMC more rapidly than Arg-AMC [24]. In this study, the results showed that Asp more rapidly hydrolyzed Leu-AMC than Arg-AMC, as well as the recombinant M28AP (Figure 2B). These results aligned with the secreted proteomics analysis, which proved that Asp contain the M28AP, because the recombinant M28AP hydrolyzed Leu-AMC, which matches the Asp hydrolyzed Leu-AMC subtracts [25].

The normal pH range of human tears is 6.5 to 7.6; the mean value is 7.0 [32]. The normal human blood plasma pH value is 7.4 [33]. The adaptation to pH allows for human pathogens to invade the tissues and bloodstream to cause severe infections [34]. AK and GAE are severe sight-threatening infections that result from the free-living pathogenic protozoans *Acanthamoeba* spp. [1,2,5]. Recombinant M28AP showed aminopeptidase activity in the approximately optimal pH range (pH 7.0–9.0), with maximum activity at pH 8.0 (Figure 2C). The human blood plasma and tears may act as suitable buffer solutions for the *Acanthamoeba* spp. to secrete the active M28AP. Hence, we suggested that the M28AP has high virulence to cause host different damage, such as complement degradation in these buffer solutions, not only human tear, but blood plasma.

### 3.2. Cell Line Apoptosis by M28AP

In this study, the CPE assay results showed that Asp of *Acanthamoeba* spp. could disrupt the co-incubated corneal epithelial cells (Figure 1A_c). A previous study showed that the M20/M25/M40 superfamily aminopeptidase protein, an aminopeptidase that is found in Asp, causes cell line disruption after *Acanthamoeba* spp. and C6 cell co-culturing [24]. In general, the cell line causes organelle stress and induces apoptosis pathways due to parasite infection [35]. Another study showed that the two main apoptotic pathways are the extrinsic and intrinsic pathways [36]. These pathways are activated by the cleavage of caspase-3, causing cell DNA fragmentation, the formation of apoptotic bodies, and the degradation of cytoskeletal and nuclear proteins [37]. Mannose-induced protein 133 (MIP 133), proteases secreted by *Acanthamoeba* spp., causes contact-independent cytolysis of corneal epithelial cells, and is able to penetrate corneal tissue [38]. Soluble metabolites that are released by *Acanthamoeba* spp. lead to human monocyte cell death through apoptosis [23]. In general, phosphatidylserine is hidden within the plasma membrane on the cytoplasmic face of the membrane. However, during cell apoptosis, phosphatidylserine is translocated to the cell surface. Annexin V is a Ca^2+^-dependent protein that has high affinity for phosphatidylserine, so it plays a role as a probe for detecting apoptosis. Propidium iodide (PI) is a fluorescent dye that binds the DNA through the membrane of late apoptotic cells and dead cells. Our results showed that C6 cells are both Annexin V- and PI-positive, because the recombinant M28AP was delivered into the individual cell line (Figure 4). The cells that were treated with the recombinant M28AP were in late apoptosis or already dead. Hence, these results showed that M28AP that was secreted by *Acanthamoeba* spp. induced the apoptosis of cell line.

## 4. Materials and Methods

### 4.1. Culture of Acanthamoeba Strains

ATCC_30010, non-pathogenic Acanthamoeba strain, were axenically cultured in protease peptone-yeast extract-glucose (PYG) medium (pH 6.5) at 28 °C in cell culture T75 flasks [39]. Trophozoites were harvested in the logarithmic growth phase after cultivation for 3–5 days to 80%. The *Acanthamoeba* cell passaging was washed three times and then resuspended in Page’s modified Neff’s amoeba saline (PAS) buffer [40].

### 4.2. Culture of C6 Glioma Rat Cell Line, A549 Human Cell Line and Human Primary Corneal Epithelial Cells

C6 glioma rat cell line and A549 human cell line were axenically cultured in Dulbecco’s Modified Eagle Medium (DMEM) High Glucose medium (Gibco^TM^, Thermo, Waltham, MA, USA) at 37 °C with 5% CO_2_ in cell culture flasks. Cell passaging was washed in phosphate buffered saline (PBS). Human primary corneal epithelial cells were axenically cultured in the EpiGRO™ Human Epidermal Keratinocyte Complete Culture Media Kit (Merck Millipore, Darmstadt, Germany).

### 4.3. Isolation of Secreted Proteins

*Acanthamoeba* spp., at an approximate density of 1.0 × 10^6^ parasites/mL, were washed three times and resuspended in PAS buffer for 4 h, after which the parasites were removed by centrifugation. The cell-free media containing secreted proteins was filtered through an Amicon^®^ Ultra-4 3K Centrifugal Filter Unit (Merck Millipore, Darmstadt, Germany) and then collected by centrifugation at 959× *g* for 75 min [24]. The protein concentration was measured with an ND-1000 (NanoDrop, Thermo, Waltham, MA, USA) and a protein assay (Bio-Rad, Hercules, CA, USA).

### 4.4. Cytopathic Effect Assay (CPE) and Giemsa Stain

Human primary corneal epithelial cells were axenically cultured in an EpiGRO™ Human Epidermal Keratinocyte Complete Culture Media Kit (Merck Millipore, Darmstadt, Germany). The human primary corneal epithelial cells were cultured to 3 × 10^5^ cells/mL in 24-well plate cell culture dishes. After 24 h of growth, *Acanthamoeba* live cells at a concentration of 3 × 10^5^ were co-cultured with human primary corneal epithelial cells. *Acanthamoeba* spp., at a density of 1.0 × 10^6^ parasites/mL in PBS for 4 h, as well as their secreted proteins, were collected. The *Acanthamoeba*-secreted proteins (150 μg) were co-incubated with human primary corneal epithelial cells for 2 h, followed by microscopy observations and CPE assays. The cells were fixed in wells with methanol with acetic acid at 3:1 for 15 min. After air drying, we added 1 mL Giemsa Buffer:Giemsas Azur-Eosin-Methylenblaulosung stain buffer (9:1) in a fixed well for 15 min. The wells were rinsed three times with ddH_2_O and then left to air dry.

### 4.5. Total RNA Isolation and cDNA Synthesis

We used a Total RNA Extraction Miniprep System (VIOGENE, Taiwan) to isolate RNA. High Capacity cDNA Reverse Transcription Kits (Thermo, Waltham, MA, USA) were used in this study. The kit components were allowed to thaw on ice. The reverse transcription conditions were set at the following times and temperatures: 25 °C for 10 min, 37 °C for 120 min, and 85 °C for 5 min; finally, the cDNA was stored at 4 °C. The reaction volume was 20 μL.

### 4.6. DNA Construction, Cell Culture and Transient Transfection

The M28AP sequence was amplified while using PCR with *Acanthamoeba* spp. cellular cDNA as the template. Forward M28AP_clon_BsrGI_F (5′-GGA TGT ACA GCA TGG TCG CTC ACG GAA GG-3′) and reverse M28AP_clon_EcoRI_stop_His_R (5′-CCG GAA TTC TCA ATG GTG ATG GTG ATG GTG CAG AGG ATC GGC GAA-3′) primers were used to amplify the cDNA encoding M28AP. The cleavage sites for the BsrGI and EcoRI restriction enzymes were incorporated at the 5′ ends of the primers for cloning into the pcDNA3.1/Zeo expression plasmid (Thermo, Waltham, MA, USA). A ratio of 1.0 μg plasmid DNA/mL of transfection culture volume was used for ExpiCHO transfection and transfection was performed at the 100 mL scale in 500 mL non-baffled flasks. The cultures were maintained at the indicated rpm on a shaker with a 19 mm throw. Cell culture and transient transfection were accomplished by following previously described protocols with minor modifications [41]. The ExpiCHO-S cells were cultured in ExpiCHO Expression medium (Thermo, Waltham, MA, USA) in a humidified 8% CO_2_ incubator at 36.5 °C and 125 rpm. Cells were split to 1~2 × 10^6^ cells/mL in each well the day prior to transfection. After 48 h, cells were >10 × 10^6^ cells/mL and they were diluted to 6.5 × 10^6^ cells/mL with the addition of fresh ExpiCHO expression medium. ExpiCHO transfection was performed using the ExpiCHO Expression System Kit (Thermo, Waltham, MA, USA), according to the manufacturer’s protocol. Briefly, ExpiFectamine transfection reagent and M28AP plasmid were separately diluted in OptiPRO SFM (Thermo, Waltham, MA, USA). ExpiFectamine and DNA mixtures were immediately combined and we waited for up to 5 min. The ExpiFectamine/plasmid complexes were then added to the cells. For transfection completed with the Max Titer Protocol, enhancer and 8% *v/v* feed were added 22~24 h post-transfections; the cultures were then temperature shifted to 32 °C and an additional 8% *v/v* feeds were added on days 3, 5, and 7 post-transfection. ExpiCHO Max Titer Protocol transfection was harvested on day 11 post-transfection. The cell cultures were centrifuged at 5000 rpm for 15 min at 4 °C and the supernatants were collected for purification.

### 4.7. SDS-PAGE and Coomassie Blue Staining

All of the protein samples were separated with 12% SDS-PAGE (T-Pro, Taiwan). SDS-PAGE running buffer consists of 3.0 g of Tris base, 14.4 g of glycine, and 1.0 g of SDS in 1000 mL of H_2_O. Following electrophoresis, the gels were placed in a solution of 50% methanol and 30% acetic acid containing distilled water for 2 h and were then placed in a solution of 30% methanol containing distilled water for 30 min to remove the acetic acid. Coomassie Blue stain was added to the gel until the protein band was detected while using a scanner (Epson, Taiwan).

### 4.8. Biochemical Properties of Recombinant Protein M28AP

The biochemical properties of recombinant protein M28AP, while using PBS buffer as the control and the activity of Asp, was assayed by the hydrolysis of L-leucine-7-amido-4-methylcoumarin hydrochloride (Leu-AMC) and l-arginine-AMC (Arg-AMC, Sigma-Aldrich, st. Louis, MO, USA). We added approximately 1 μg/mL the recombinant protein M28AP solution to 195 μL of assay buffer (50 mM Tris-HCl, pH 8.0) containing 10 μM Leu-AMC or Arg-AMC up to 200 μL, which was then incubated for 30 min at 37 °C. The release of fluorescence was measured at an excitation wavelength of 370 nm and an emission wavelength of 440 nm while using a FlexStation 3 Multi-Mode microplate reader (Molecular Devices, Sunnyvale, CA, USA) [21].

### 4.9. Ni-NTA-Atto Conjugates for Recombinant Protein M28AP- Cell Line Interaction

Ni-NTA-Atto conjugates provide specific and highly sensitive detection of His-tagged fusion proteins. The Ni-NTA-Atto conjugates were incubated with recombinant protein M28AP (50 μg) at 37 °C for 30 min. After incubation, the Ni-NTA-Atto-M28AP complex was added to C6 and A549 cells for 4 h. After washing three times with PBS, a Cell^R^ microscope (Olympus Cell^R^, Tokyo, Japan) was used for light and fluorescence microscopy. Image analysis was performed with Cell^R^ software (Olympus Cell^R^, Tokyo, Japan).

### 4.10. Annexin V Apoptosis Assay

C6 cells, with an approximate density of 1.0 × 10^5^ cells/mL, were seeded overnight. Recombinant protein M28AP (14 μg) was added to C6 cells at 37 °C for 24 h. Annexin V-FAM + PI Apoptosis Detection Reagent (Leadgen, Taiwan) were used. We mixed 5 μL of Annexin V- FAM and 5 μL of PI with 500 μL binding buffer to C6 cells, which were then gently mixed and incubated for 15~20 min. at 37 °C in the dark. After washing three times with PBS, a Cell^R^ microscope (Olympus Cell^R^, Tokyo, Japan) was used for light and fluorescence microscopy. Cell^R^ software was used to analyze the images.
